# Supplementary material for: Anti-PD-1 cis-delivery of low-affinity IL-12 activates intratumoral CD8+T cells for systemic antitumor responses
Source: Nat Commun. 2024 Jun 3;15:4701. doi: 10.1038/s41467-024-49034-1 (PMC11148143; doi:10.1038/s41467-024-49034-1)
Supplement: Supplementary file 1 — Supplementary Information [file 41467_2024_49034_MOESM1_ESM.pdf]

Anti-PD-1 *cis*-delivery of low-affinity IL-12 activates intratumoral CD8⁺T cells for systemic antitumor responses

S1

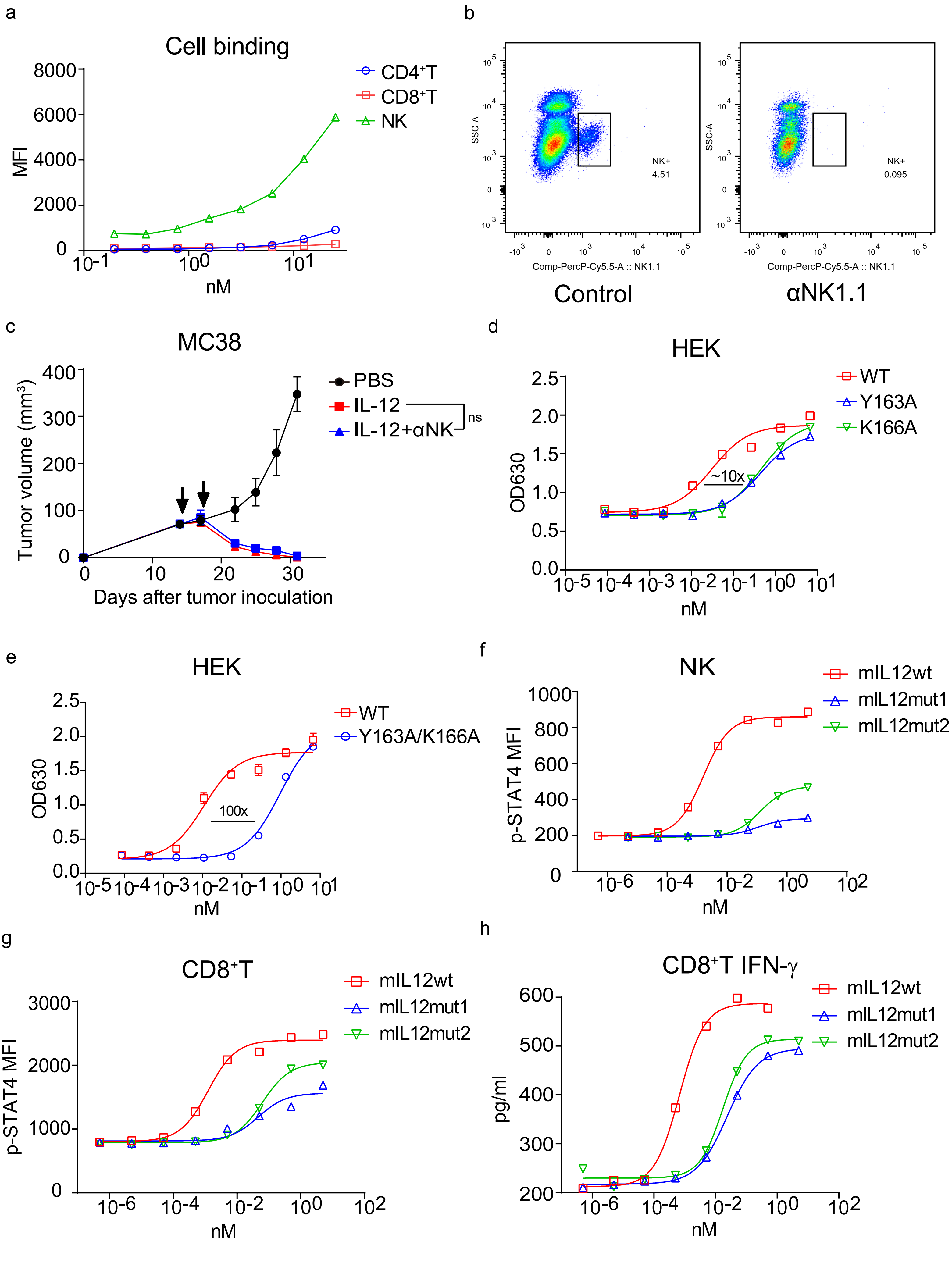

Supplementary Fig. 1 Both IL-12 mutants displayed attenuated bioactivities

(a) Splenocytes were incubated with IL-12 *in vitro*. Protein binding to $CD4^{+}$T cells, $CD8^{+}$T cells, or NK cells was detected by flow cytometric analysis. (b, c) MC38 tumor-bearing mice (n = 4) were intraperitoneally treated with PBS or 5μg IL-12 on days 14 and 17. For NK cell depletion, mice were intraperitoneally injected with 400μg αNK1.1 antibody one day before treatment and then once every 3 days for three times. (b) The NK cells depletion efficacy. (c) The tumor growth curve. (d) The activity of Y163A or K166A mutant IL-12 was detected using HEK reporter cells. (e) The activity of Y163A/K166A mutant IL-12 was detected using HEK reporter cells. (f) NK cells were incubated with mIL12wt, mIL12mut1, or mIL12mut2 for 0.5h *in vitro*. The p-STAT4 in NK cells was detected by flow cytometric analysis. (g) Pre-activated $CD8^{+}$T cells were incubated with mIL12wt, mIL12mut1, or mIL12mut2 for 0.5h *in vitro*. The p-STAT4 in $CD8^{+}$T cells was detected by flow cytometric analysis. (h) Pre-activated $CD8^{+}$T cells were incubated with mIL12wt, mIL12mut1, or mIL12mut2 for 48h. The IFN-γ in the supernatant was detected. Data are shown as mean ± SD from two to three independent experiments. The P value was determined by two-way ANOVA (c).

S2

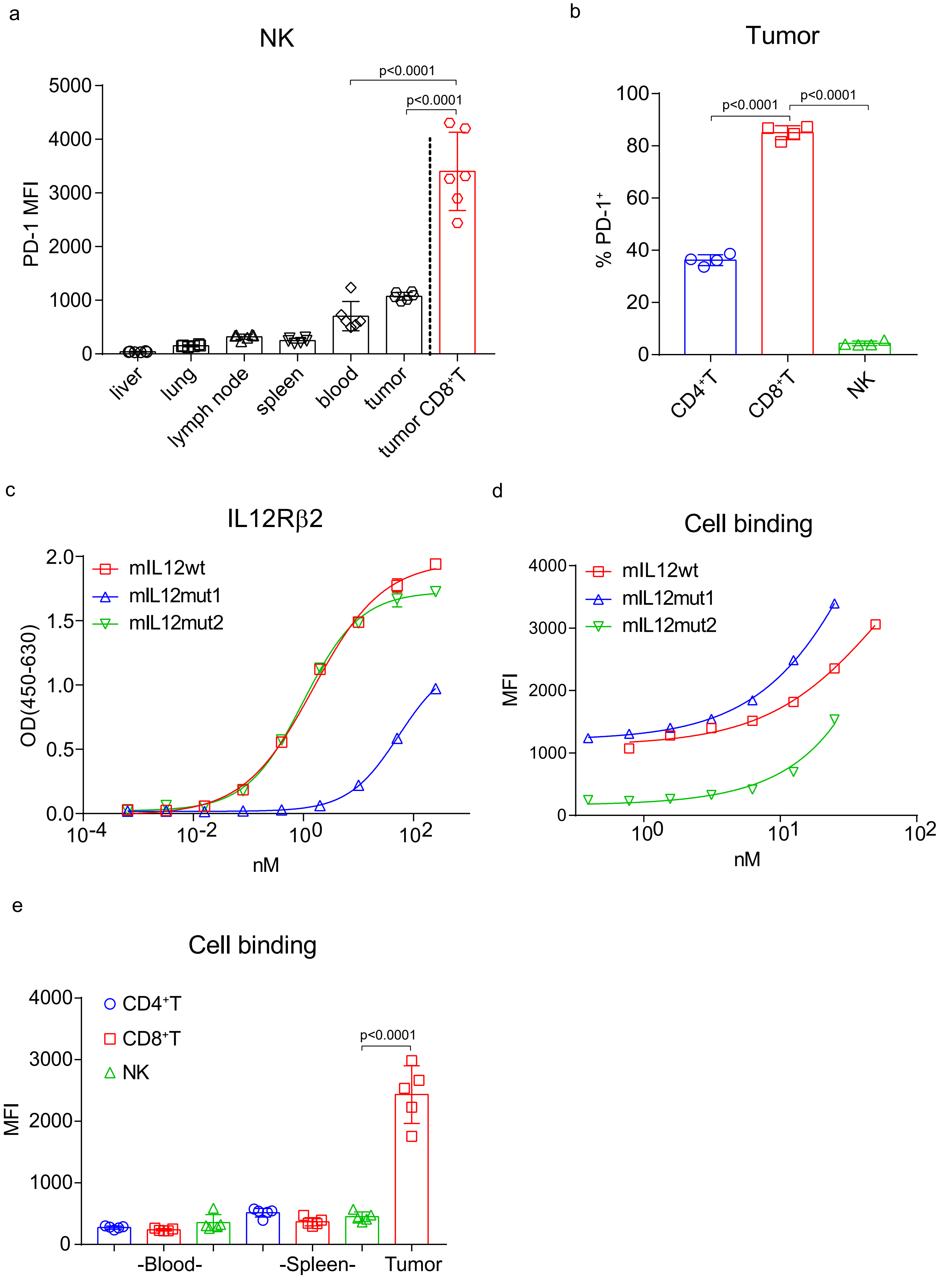
a
NK
p<0.0001
p<0.0001
PD-1 MFI
5000
4000
3000
2000
1000
0
liver
lung
lymph node
spleen
blood
tumor
tumor CD8+T
b
Tumor
p<0.0001
p<0.0001
% PD-1+
100
80
60
40
20
0
CD4+T
CD8+T
NK
c
IL12Rβ2
mIL12wt
mIL12mut1
mIL12mut2
OD(450-630)
2.0
1.5
1.0
0.5
0.0
10-4
10-2
100
102
nM
d
Cell binding
mIL12wt
mIL12mut1
mIL12mut2
MFI
4000
3000
2000
1000
0
100
101
102
nM
e
Cell binding
CD4+T
CD8+T
NK
p<0.0001
MFI
4000
3000
2000
1000
0
-Blood-
-Spleen-
Tumor

Supplementary Fig. 2 Anti-PD1-mIL12mut2 targeted tumor tissue

(a) The PD-1 expression on NK cells in different tissues from MC38 tumor-bearing mice (n = 6) compared to the intratumoral $CD8^{+}T$ cells. (b) The frequency of $PD\text{-}1^{+}CD8^{+}T$ cells in MC38 tumor (n = 4). (c) The binding curve of mIL12wt, mIL12mut1, or mIL12mut2 with IL-12Rβ2 was measured by ELISA. (d) Pre-activated T cells were incubated with serially diluted mIL12wt, mIL12mut1, or mIL12mut2. Protein binding to T cells was detected by flow cytometric analysis. (e) The single-cell suspension from blood, spleen, or MC38 tumor was incubated with αPD1-mIL12mut2 *in vitro*. Protein binding to $CD4^{+}T$ cells, $CD8^{+}T$ cells, or NK cells was detected by flow cytometric analysis (n = 5 mice). Data are shown as mean ± SD from two to three independent experiments. The P value was determined by one-way ANOVA (a, b, e).

S3

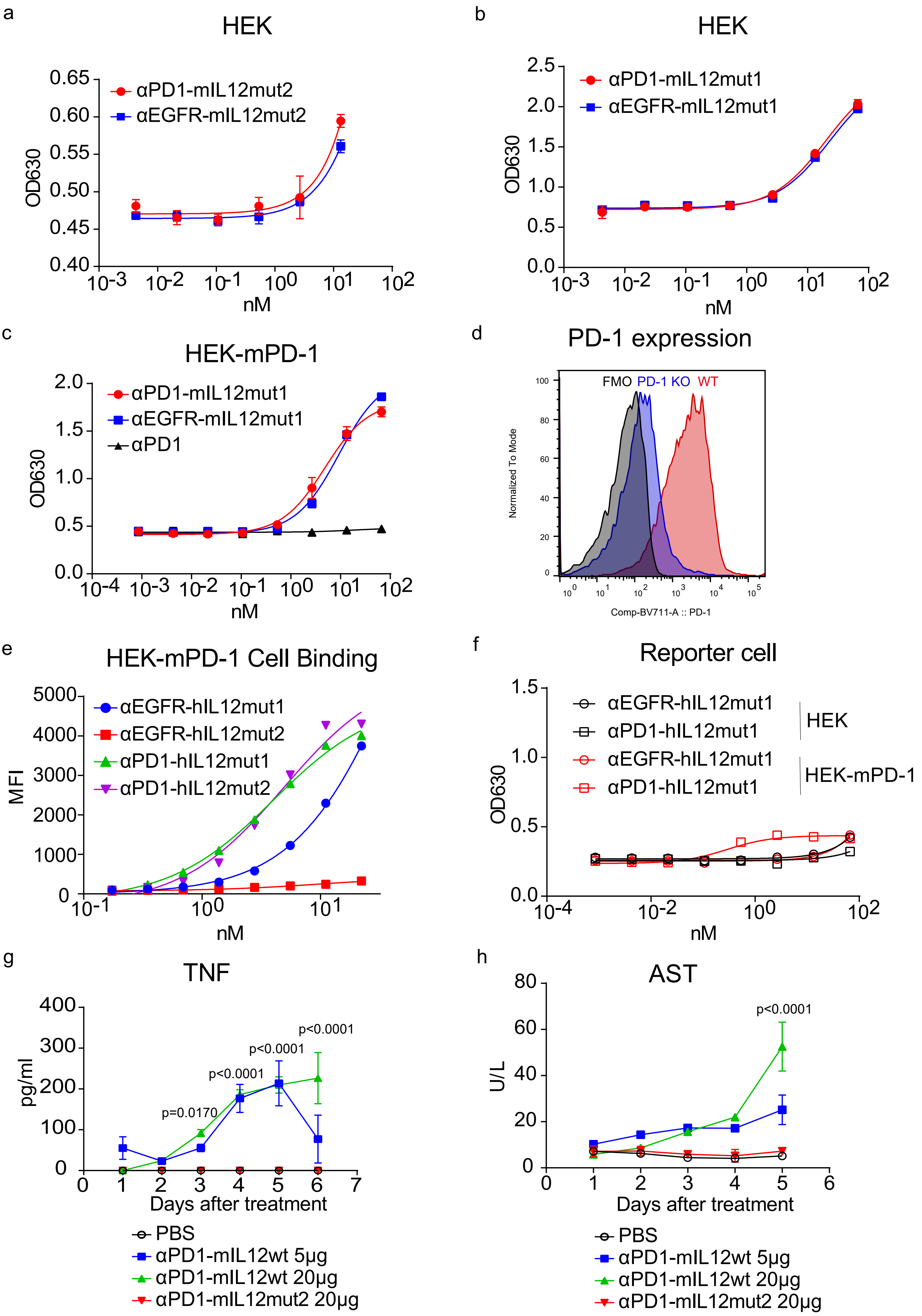

a
HEK
0.65
0.60
0.55
0.50
0.45
0.40
OD630
αPD1-mIL12mut2
αEGFR-mIL12mut2
10-3 10-2 10-1 100 101 102
nM
b
HEK
2.5
2.0
1.5
1.0
0.5
0.0
OD630
αPD1-mIL12mut1
αEGFR-mIL12mut1
10-3 10-2 10-1 100 101 102
nM
c
HEK-mPD-1
2.0
1.5
1.0
0.5
0.0
OD630
αPD1-mIL12mut1
αEGFR-mIL12mut1
αPD1
10-4 10-3 10-2 10-1 100 101 102
nM
d
PD-1 expression
FMO
PD-1 KO
WT
100
80
60
40
20
0
Normalized To Mode
10^0 10^1 10^2 10^3 10^4 10^5
Comp-BV711-A :: PD-1
e
HEK-mPD-1 Cell Binding
5000
4000
3000
2000
1000
0
MFI
αEGFR-hIL12mut1
αEGFR-hIL12mut2
αPD1-hIL12mut1
αPD1-hIL12mut2
10-1 100 101
nM
f
Reporter cell
1.5
1.0
0.5
0.0
OD630
αEGFR-hIL12mut1
αPD1-hIL12mut1
HEK
αEGFR-hIL12mut1
αPD1-hIL12mut1
HEK-mPD-1
10-4 10-2 100 102
nM
g
TNF
400
300
200
100
0
pg/ml
p=0.0170
p<0.0001
p<0.0001
p<0.0001
0 1 2 3 4 5 6 7
Days after treatment
PBS
αPD1-mIL12wt 5μg
αPD1-mIL12wt 20μg
αPD1-mIL12mut2 20μg
h
AST
80
60
40
20
0
U/L
p<0.0001
0 1 2 3 4 5 6
Days after treatment
PBS
αPD1-mIL12wt 5μg
αPD1-mIL12wt 20μg
αPD1-mIL12mut2 20μg

Supplementary Fig. 3 Anti-PD-1 mediated binding cannot restore the activity of mIL12mut1 on HEK-mPD-1 cells

(a) The activity of αEGFR-mIL12mut2 or αPD1-mIL12mut2 was detected using the HEK reporter cells. (b) The activity of αEGFR-mIL12mut1 or αPD1-mIL12mut1 was detected using the HEK reporter cells. (c) The activity of αEGFR-mIL12mut1 or αPD1-mIL12mut1 was detected using the HEK-mPD-1 cells. (d) The PD-1 expression on $CD8^{+}$T cells from WT or PD-1 KO mice after *in vitro* activation. (e) HEK-mPD-1 cells were incubated with αEGFR-hIL12mut1, αEGFR-hIL12mut2, αPD1-hIL12mut1, or αPD1-hIL12mut2 *in vitro*. Protein binding to HEK-mPD-1 cells was detected by flow cytometric analysis. (f) The activity of αEGFR-hIL12mut1 or αPD1-hIL12mut1 was detected using the HEK or HEK-mPD-1 cells. (g, h) MC38 tumor-bearing mice (n = 3) were intraperitoneally treated once with PBS or 5μg αPD1-mIL12wt or 20μg αPD1-mIL12wt or 20μg αPD1-mIL12mut2. After treatment, (g) TNF and (h) AST in serum were measured. Data are shown as mean ± SD from two to three independent experiments. The P value was determined by two-way ANOVA (g, h).

S4

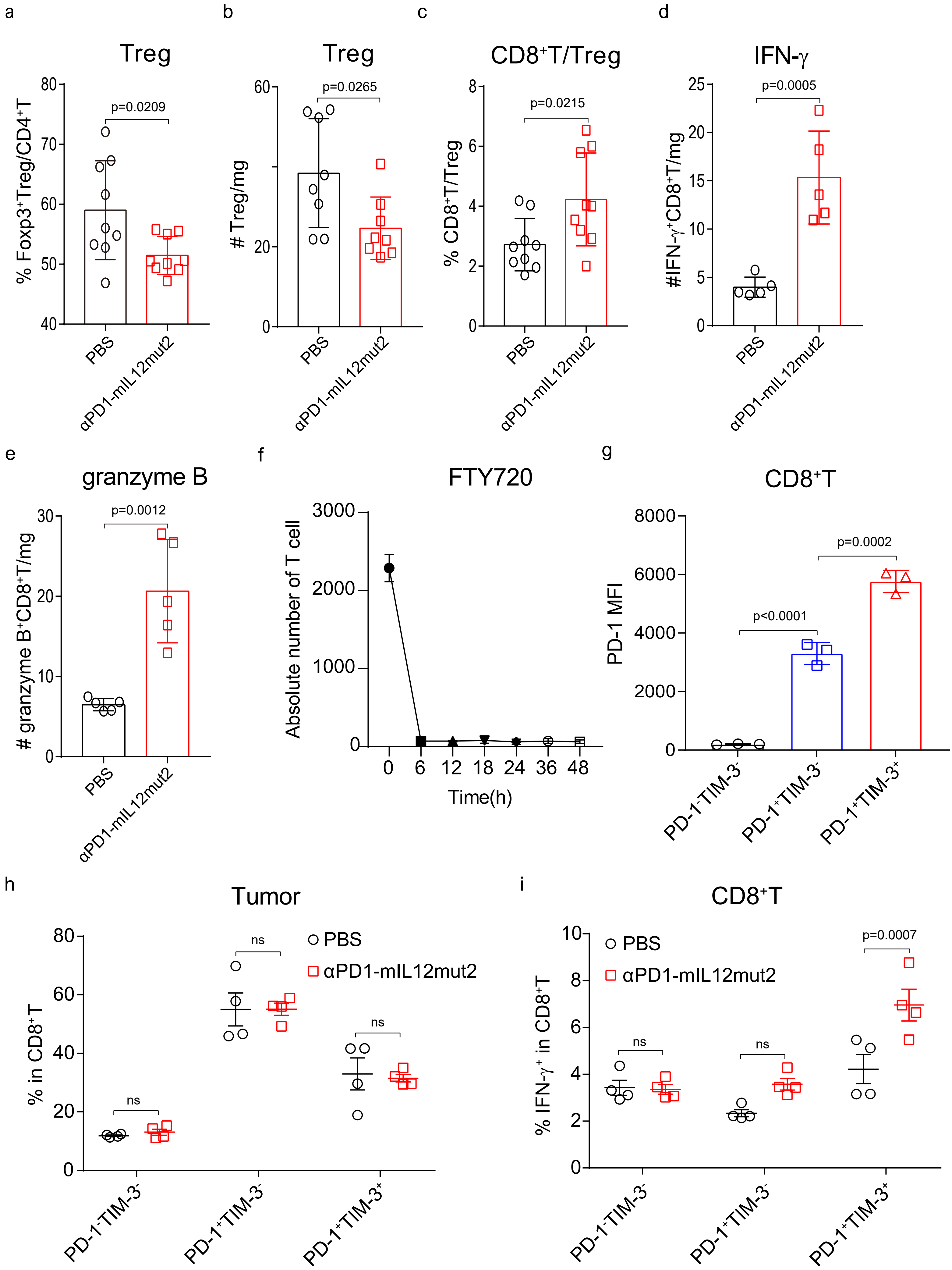

a
Treg
% Foxp3+Treg/CD4+T
p=0.0209
PBS
αPD1-mIL12mut2
b
Treg
# Treg/mg
p=0.0265
c
CD8+T/Treg
% CD8+T/Treg
p=0.0215
d
IFN-γ
#IFN-γ+CD8+T/mg
p=0.0005
e
granzyme B
# granzyme B+CD8+T/mg
p=0.0012
f
FTY720
Absolute number of T cell
Time(h)
g
CD8+T
PD-1 MFI
p=0.0002
p<0.0001
PD-1-TIM-3-
PD-1+TIM-3-
PD-1+TIM-3+
h
Tumor
% in CD8+T
ns
PBS
αPD1-mIL12mut2
i
CD8+T
% IFN-γ+ in CD8+T
p=0.0007

Supplementary Fig. 4 Anti-PD1-mIL12mut2 preferentially activated PD-1^+TIM-3^+CD8^+T cells in tumors (a-c) MC38 tumor-bearing mice (n = 8-9) were intraperitoneally treated twice with PBS or 20μg αPD1-mIL12mut2. 48 hours after the second treatment, the lymphocytes in the tumor were analyzed. (A) The frequency of Foxp3^+Treg cells in CD4^+T cells. (b) The number of Treg cells. (c) The ratio of CD8^+T/Treg. (d) The number of IFN-γ^+CD8^+T cells after αPD1-mIL12mut2 treatment in tumor (n = 5 mice/group). (e) The number of granzyme B^+CD8^+T cells after αPD1-mIL12mut2 treatment in tumor (n = 5 mice/group). (f) Tumor-bearing mice (n = 4) were intraperitoneally injected with 25μg FTY720, and the blood samples were collected at different time points after FTY720 treatment. The absolute number of CD3^+T cells in the blood was detected. (g) The PD-1 expression on different intratumoral CD8^+T cell subsets in the MC38 tumor (n = 4 mice). (h) The frequency of different intratumoral CD8^+T cell subsets after αPD-1-IL-12m treatment (n = 4 mice). (i) MC38 tumor-bearing mice (n = 4 mice/group) were intratumorally treated once with PBS or 10μg αPD1-mIL12mut2. The frequency of IFN-γ^+ cells in different intratumoral CD8^+T cell subsets was detected by *in vivo* intracellular cytokine staining Data are shown as mean ± SD from two to three independent experiments. The P value was determined by unpaired *t* tests (a-e, g) or two-way ANOVA (h, i).

S5

a

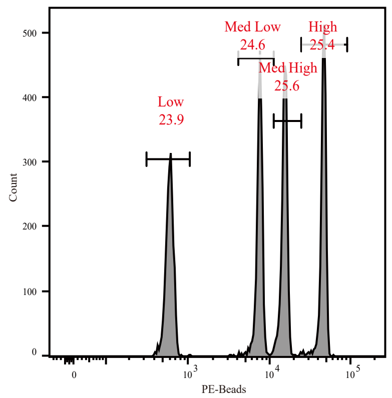

b

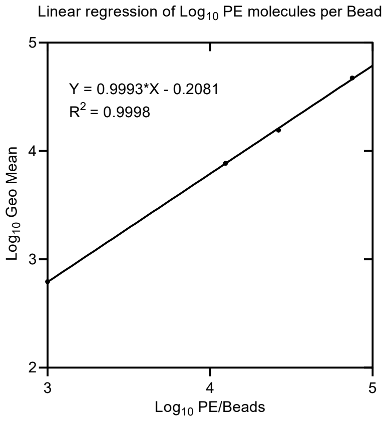

c

Marker	Events	Geometric Means	Lg Geometric Means	PE Molecules/Bead	Lg PE Molecules/Bead
All	9407	7146	Y value		X value
Low	2265	621	2.793	1000	3
Med Low	2290	7691	3.886	12445	4.095
Med High	2410	15602	4.193	26427	4.422
High	2379	47309	4.675	74906	4.875

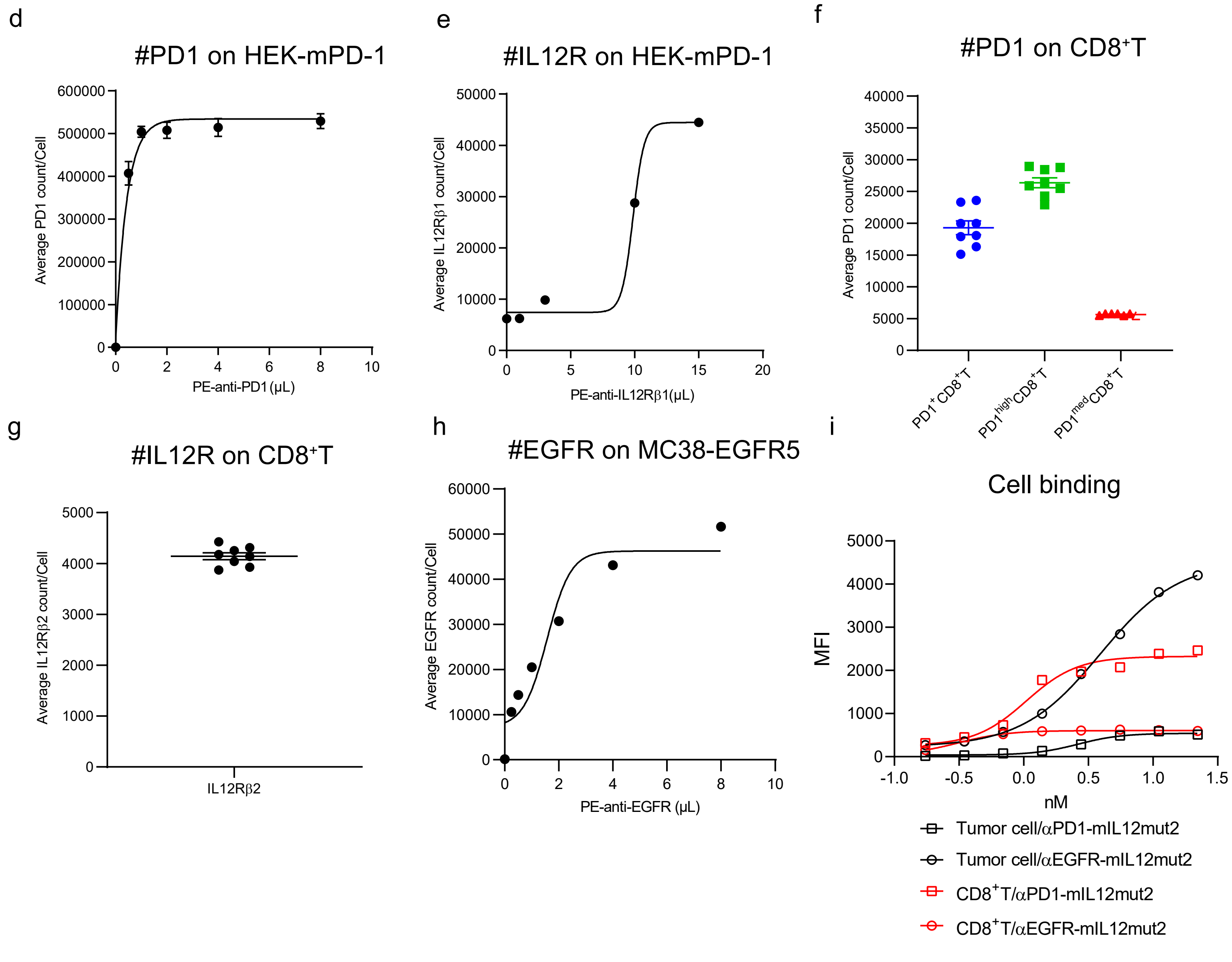

Supplementary Fig. 5 Receptors qualification

(a) Distinctly separated four-peak signals of PE beads were illustrated on a PE spectrum. The four peaks corresponded to four beads carrying varying numbers of PE and were categorized as Low, Med Low, Med High, and High. (b) The standard curve was derived through linear regression of Log10 PE/Bead and Log10 Geo Mean, following the equation: $Y = 0.9993*X - 0.2081$, where Y represents Log10 Geo Mean and X represents Log10 PE/Bead. (c) Statistics of Geometric Mean and PE molecule quantities of the four PE beads. Geometric Mean was assessed by flow cytometry, and data on PE Molecules per Bead was gathered following the kit instructions. Log10 Geometric Mean and Log10 PE Molecules/Bead were calculated and plotted for the standard curve. (d, e) The PE-anti-PD1 and PE-anti-IL12Rβ1 antibody were saturated at 8 μL and 15 μL respectively within a non-linear fitting curve with increasing titration of the PE antibody. The Geo Means of PE-labeled anti-PD1 and anti-IL12Rβ1 were substituted into the equation to calculate mean PD1 (d) and IL12Rβ1 (e) counts per cell. (f, g) C57BL/6 mice (n = 8) were inoculated with 5×10^5 MC38 tumor cells. On day 14, MC38 tumors were collected and processed. The Geo Means of PE-labeled anti-PD1 and anti-IL12Rβ2 binding to the intratumoral $CD8^+$T cells were identified via flow cytometry. The mean count of PD1 (f) or IL12Rβ2 (g) on $CD8^+$T cells was quantified at the saturation concentration according to the fitting curve. (h) The PE-anti-EGFR antibody was saturated at 8 μL within a non-linear fitting curve with increasing titration of PE antibody. The Geo Means were substituted into the equation to determine the mean EGFR counts per cell. (i) Digested tumor tissues from the MC38-EGFR5 tumors were incubated with αEGFR-mIL12mut2 or αPD1-mIL12mut2. Fusion protein binding to the tumor cells or $CD8^+$T cells was detected by flow cytometric analysis. All data are shown as mean ± SD from two to three independent experiments.

S6

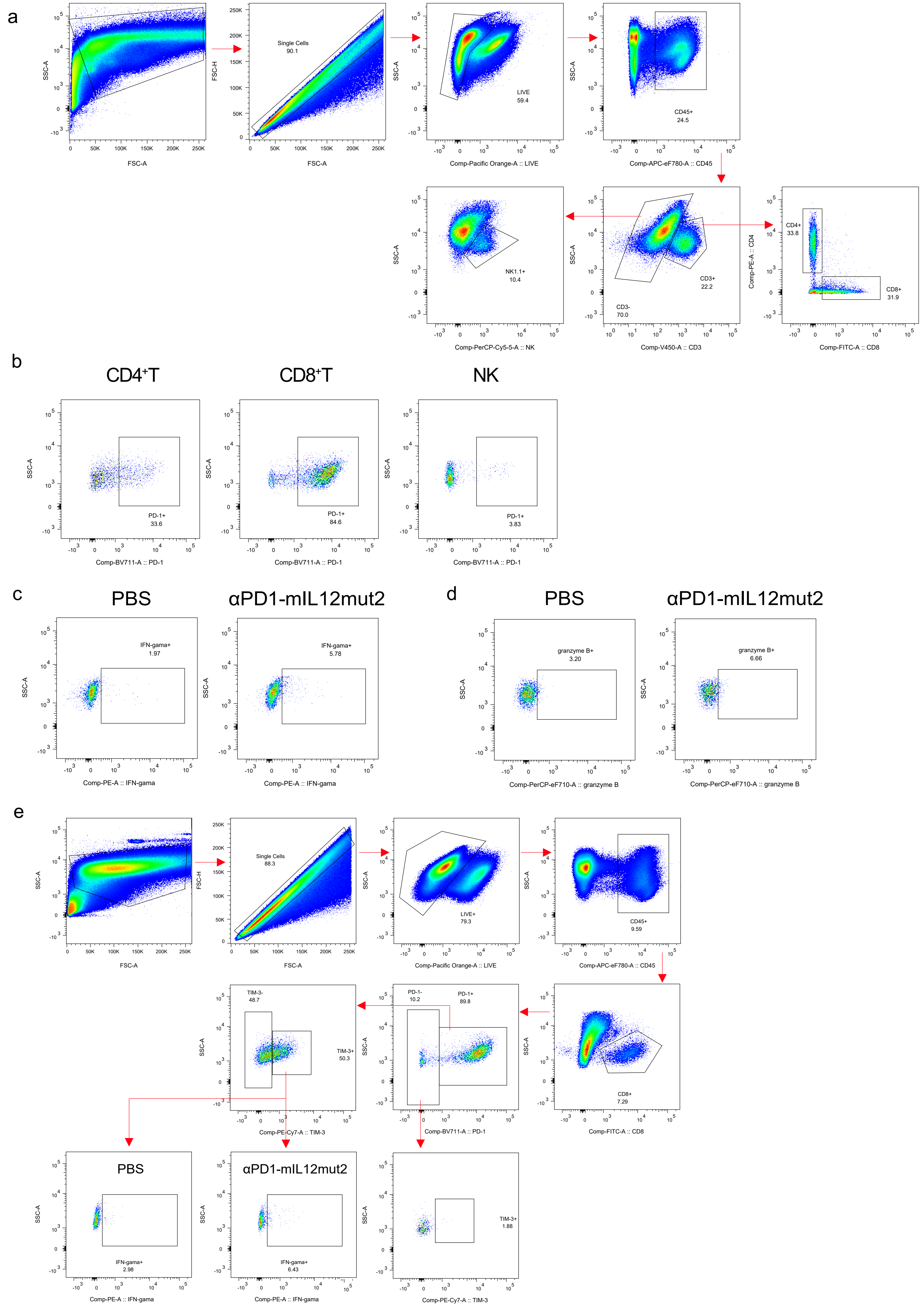
a
SSC-A
FSC-A
FSC-H
Single Cells
90.1
Comp-Pacific Orange-A :: LIVE
LIVE
59.4
Comp-APC-eF780-A :: CD45
CD45+
24.5
Comp-PerCP-Cy5-5-A :: NK
NK1.1+
10.4
Comp-V450-A :: CD3
CD3+
22.2
CD3-
70.0
Comp-PE-A :: CD4
CD4+
33.8
Comp-FITC-A :: CD8
CD8+
31.9
b
CD4+T
CD8+T
NK
Comp-BV711-A :: PD-1
PD-1+
33.6
PD-1+
84.6
PD-1+
3.83
c
PBS
αPD1-mIL12mut2
IFN-gama+
1.97
IFN-gama+
5.78
Comp-PE-A :: IFN-gama
d
PBS
αPD1-mIL12mut2
granzyme B+
3.20
granzyme B+
6.66
Comp-PerCP-eF710-A :: granzyme B
e
Single Cells
88.3
LIVE+
79.3
CD45+
9.59
CD8+
7.29
PD-1-
10.2
PD-1+
89.8
Comp-BV711-A :: PD-1
TIM-3-
48.7
TIM-3+
50.3
Comp-PE-Cy7-A :: TIM-3
PBS
αPD1-mIL12mut2
IFN-gama+
2.98
IFN-gama+
6.43
TIM-3+
1.88

Supplementary Fig. 6 The gating strategies

(a) The gating strategy for $CD4^+T$, $CD8^+T$ and NK cells from MC38 tumors. This strategy was also used for $CD4^+T$, $CD8^+T$ and NK cells gating from liver, spleen, lung, kidney, and blood, related to Fig. 2a and supplementary Fig. 2a. (b) Using the gating strategy depicted in (a), the PD-1 positive cells were gated in $CD4^+T$, $CD8^+T$, and NK cells, related to Fig. 2b and supplementary Fig. 2b. (c) The IFN-γ positive cells were gated in $CD8^+T$ cells in tumors after PBS or αPD1-mIL12mut2 treatment, related to Fig. 5c. (d) The granzyme-B positive cells were gated in $CD8^+T$ cells in tumors after PBS or αPD1-mIL12mut2 treatment, related to Fig. 5d. (e) The gating strategy for $PD\text{-}1^-$, $PD\text{-}1^+TIM\text{-}3^-$, and $PD\text{-}1^+TIM\text{-}3^+CD8^+T$ cells from MC38 tumors, related to supplementary Fig. 4h. The IFN-γ positive cells were gated in $PD\text{-}1^+TIM\text{-}3^+CD8^+T$ cells in tumors after PBS or αPD1-mIL12mut2 treatment, related to supplementary Fig. 4i.
