# Supplementary material for: Anti-PD-1 cis-delivery of low-affinity IL-12 activates intratumoral CD8+T cells for systemic antitumor responses
Source: Nat Commun. 2024 Jun 3;15:4701. doi: 10.1038/s41467-024-49034-1 (PMC11148143; doi:10.1038/s41467-024-49034-1)
Supplement: Supplementary file 3 — Reporting Summary [file 41467_2024_49034_MOESM3_ESM.pdf]

nature portfolio

Corresponding author(s): Yang-Xin Fu and Hua Peng

Last updated by author(s): Apr 24, 2024

Reporting Summary

Nature Portfolio wishes to improve the reproducibility of the work that we publish. This form provides structure for consistency and transparency in reporting. For further information on Nature Portfolio policies, see our Editorial Policies and the Editorial Policy Checklist.

Statistics

For all statistical analyses, confirm that the following items are present in the figure legend, table legend, main text, or Methods section.

n/a	Confirmed	
[ ]	[x]	The exact sample size (*n*) for each experimental group/condition, given as a discrete number and unit of measurement
[ ]	[x]	A statement on whether measurements were taken from distinct samples or whether the same sample was measured repeatedly
[ ]	[x]	The statistical test(s) used AND whether they are one- or two-sided<br>*Only common tests should be described solely by name; describe more complex techniques in the Methods section.*
[x]	[ ]	A description of all covariates tested
[x]	[ ]	A description of any assumptions or corrections, such as tests of normality and adjustment for multiple comparisons
[ ]	[x]	A full description of the statistical parameters including central tendency (e.g. means) or other basic estimates (e.g. regression coefficient) AND variation (e.g. standard deviation) or associated estimates of uncertainty (e.g. confidence intervals)
[ ]	[x]	For null hypothesis testing, the test statistic (e.g. *F*, *t*, *r*) with confidence intervals, effect sizes, degrees of freedom and *P* value noted<br>*Give P values as exact values whenever suitable.*
[x]	[ ]	For Bayesian analysis, information on the choice of priors and Markov chain Monte Carlo settings
[x]	[ ]	For hierarchical and complex designs, identification of the appropriate level for tests and full reporting of outcomes
[x]	[ ]	Estimates of effect sizes (e.g. Cohen's *d*, Pearson's *r*), indicating how they were calculated

Our web collection on statistics for biologists contains articles on many of the points above.

Software and code

Policy information about availability of computer code

Data collection	FACS Fortessa (BD Biosciences); SpectraMax Plus 384 (Molecular Devices)
Data analysis	FACS data: FlowJo v10.8.1 (Treestar); GraphPad Prism v8.0.1; Microsoft Excel 2019; Adobe Illustrator 2021.

For manuscripts utilizing custom algorithms or software that are central to the research but not yet described in published literature, software must be made available to editors and reviewers. We strongly encourage code deposition in a community repository (e.g. GitHub). See the Nature Portfolio guidelines for submitting code & software for further information.

Data

Policy information about availability of data

All manuscripts must include a data availability statement. This statement should provide the following information, where applicable:

- Accession codes, unique identifiers, or web links for publicly available datasets
- A description of any restrictions on data availability
- For clinical datasets or third party data, please ensure that the statement adheres to our policy

The main data supporting the results in this study are available within the paper. Additional data supporting the results of the study are also available from the corresponding authors on reasonable request.

Research involving human participants, their data, or biological material

Policy information about studies with human participants or human data. See also policy information about sex, gender (identity/presentation), and sexual orientation and race, ethnicity and racism.

Reporting on sex and gender	No human participants is included.
Reporting on race, ethnicity, or other socially relevant groupings	No human participants is included.
Population characteristics	No human participants is included.
Recruitment	No human participants is included.
Ethics oversight	No human participants is included.

Note that full information on the approval of the study protocol must also be provided in the manuscript.

Field-specific reporting

Please select the one below that is the best fit for your research. If you are not sure, read the appropriate sections before making your selection.

☒ Life sciences ☐ Behavioural & social sciences ☐ Ecological, evolutionary & environmental sciences

For a reference copy of the document with all sections, see nature.com/documents/nr-reporting-summary-flat.pdf

Life sciences study design

All studies must disclose on these points even when the disclosure is negative.

Sample size	Sample sizes were predetermined from pilot experiments and/or experiments that have been done in the past, to obtain statistically significant data.
Data exclusions	No data was excluded for all experiments.
Replication	As reported in the figure legends, experiments were performed two or three times with similar results.
Randomization	For all in vivo experiments, animals were randomly assigned into a treatment group after tumor inoculation. The starting tumor burden in the treatment and control groups was similar before treatment. For all in vitro experiments, samples with the same origin were randomly allocated into control and experimental groups.
Blinding	Investigators were not blinded to group allocation during experimental setup, data collection, and analysis. No blinding was performed since we did not have the personnel resources to consistently perform blinding

Reporting for specific materials, systems and methods

We require information from authors about some types of materials, experimental systems and methods used in many studies. Here, indicate whether each material, system or method listed is relevant to your study. If you are not sure if a list item applies to your research, read the appropriate section before selecting a response.

Materials & experimental systems

n/a	Involved in the study	
☐	☒	Antibodies
☐	☒	Eukaryotic cell lines
☒	☐	Palaeontology and archaeology
☐	☒	Animals and other organisms
☒	☐	Clinical data
☒	☐	Dual use research of concern
☒	☐	Plants

Methods

n/a	Involved in the study	
☒	☐	ChIP-seq
☐	☒	Flow cytometry
☒	☐	MRI-based neuroimaging

Antibodies

Antibodies used	For in vivo depletion: Anti-CD8 antibody (TIB210), anti-CD4 antibody (GK1.5), anti-NK1.1 antibody (PK136) and anti-IFN-gamma antibody (XMG1.2) were produced in-house.

	For in vivo mice treatment: anti-mouse PD-1 (J43) and anti-human PD-1 (Keytruda) were produced in-house.<br>For FACS:<br>Anti-mCD45 (30-F11), Invitrogen, 47-0451-82;<br>Anti-mCD3 (17A2), eBioscience, 48-0032-80;<br>Anti-mCD4(GK1.5), eBioscience, 17-0041-81;<br>Anti-mCD8 (53-6.7), eBioscience, 11-0081-82;<br>Anti-mNK1.1 (PK136), eBioscience, 17-5941-81;<br>Anti-mPD-1 (29F.1A12), BioLegend,135231;<br>Anti-mTIM-3 (RMT3-23), BioLegend, 119715;<br>Anti-mIFN-γ (XMG1.2), BioLegend, 505808;<br>Anti-hIgG Fc (M1310G05), BioLegend, 410722;<br>Anti-human IL-12Rb1 (69310), R&D, FAB839P;<br>Anti-mouse IL-12Rb2 (305719), R&D, FAB1959P;<br>Anti-mouse/human p-STAT4 (Tyr693)(4LURPIE), Invitrogen, 17-9044-42;<br>Anti-mouse granzyme B (NGZB), Invitrogen, 46-8898-80;<br>Anti-mouse FoxP3 (FJK-16s), eBioscience, 17-5773-82;<br>Yellow Fluorescent reactive dye, Invitrogen, 2438368.<br><br>For ELISA: Peroxidase AffiniPure Mouse Anti-Human IgG, Fcγ fragment specific, Jackson ImmunoResearch Laboratories, 209-035-098.<br><br>For Cytometric Bead Array: anti-IFN-γ and anti-TNF, BD Cytometric Bead Array mouse inflammation Kit, 552364.
Validation	All antibodies were well-recognized clones in the field and validated by the manufacturers. Please see manufacturer websites for validation data. These antibodies are routinely used in our laboratory without additional validation.

Eukaryotic cell lines

Policy information about cell lines and Sex and Gender in Research

Cell line source(s)	MC38, B16F10, A549, and 293T cell lines were purchased from American Type Culture Collection.<br>The MC38-EGFR5 cell line was obtained from the transduction by lentivirus encoding the mutant mouse EGFR gene.<br>Freestyle 293F cell (R79007) was purchased from Invitrogen.<br>HEK-Blue™ IL-12 cell line was purchased from InvivoGen.<br>The HEK-Blue™ IL-12-mPD-1 cell line was obtained from the transduction by lentivirus encoding the mouse Pdcd1 gene and selected by puromycin.
Authentication	The cell lines from manufacturers were not authenticated. MC38-EGFR5 and HEK-Blue™ IL-12-mPD-1 cell line were authenticated by flow cytometry.
Mycoplasma contamination	All cell lines were routinely tested for mycoplasma contamination. All cells used in this study are negative for mycoplasma.
Commonly misidentified lines<br>(See ICLAC register)	No commonly misidentified cell lines were used in this study.

Animals and other research organisms

Policy information about studies involving animals; ARRIVE guidelines recommended for reporting animal research, and Sex and Gender in Research

Laboratory animals	C57BL/6 mice were purchased from SPF biotechnology. Rag1-/- mice were purchased from the Model Animal Research Center, Nanjing University. Pdcd1-/- mice were purchased from the Animal Management Center, Institute of Biophysics. NOD scid gamma mice were maintained internally. 6-8 weeks old mice were used for experiments.
Wild animals	The study did not involve wild animals.
Reporting on sex	Female mice were used in all the experiments.
Field-collected samples	No field-collection was performed.
Ethics oversight	Animal care and experiments were carried out under institutional protocol and guidelines. All studies were approved by the Animal Care and Use Committee of the Institute of Biophysics

Note that full information on the approval of the study protocol must also be provided in the manuscript.

Flow Cytometry

Plots

Confirm that:

- [x] The axis labels state the marker and fluorochrome used (e.g. CD4-FITC).
- [x] The axis scales are clearly visible. Include numbers along axes only for bottom left plot of group (a 'group' is an analysis of identical markers).
- [x] All plots are contour plots with outliers or pseudocolor plots.
- [x] A numerical value for number of cells or percentage (with statistics) is provided.

Methodology

Sample preparation	For p-STAT4 signal and IFN-γ induction: The spleen from wild-type or pdcd1-/- C57BL/6 mice were isolated. Red blood cells were lysed using ACK lysis buffer, and a single cell suspension was generated. Splenocytes were activated with 2.5μg/ml αCD3, 2.5μg/ml αCD28, and 100IU/ml recombinant IL-2 for 48h at 37°C. CD8+T cells were sorted using a mouse CD8+T cell isolation kit (BioLegend). NK cells were sorted from fresh splenocytes using a mouse NK cell isolation kit (BioLegend). For the p-STAT4 signal, CD8+T cells or NK cells were incubated with different proteins for 30 minutes at 37°C. Cells were stained with anti-STAT4 pY693 APC (4LURPIE, Invitrogen) and analyzed by Flow Cytometry. For IFN-γ induction, CD8+T cells or NK cells were incubated with different proteins for 48h at 37°C. The supernatant was assessed for IFN-γ using a CBA kit (BD).<br><br>For Cis-binding assay: Pre-activated wild-type or pdcd1-/- CD8+T cells were sorted using a CD8+T cell isolation kit (BioLegend). The wild-type CD8+T cells were labeled with Cell trace violet (CTV). An equal number of CTV-labeled wild-type CD8+T cells and unlabeled pdcd1-/- CD8+T cells were mixed and incubated with αEGFR-mIL12mut2 or αPD1-mIL12mut2 for 30 minutes at 37°C. Cells were stained with anti-p-STAT4 and analyzed by Flow Cytometry.<br><br>For Flow cytometry: Tumor tissues were collected, cut into small pieces, and re-suspended in digestion buffer (RPMI-1640 medium with 1mg/mL type IV collagenase and 100μg/mL DNase I). Tumors were digested for 45 min at 37°C and then passed through a 70-μm cell strainer to make single-cell suspensions. Single-cell suspensions were incubated with FcγR II/III blocking antibody (2.4G2) and stained with specific antibodies followed by established protocol.
Instrument	FACS Fortessa (BD Biosciences)
Software	FlowJo v10.8.1 (Treestar)
Cell population abundance	When cells were sorted, the purity was confirmed by flow cytometry and in each case was above 90% purity.
Gating strategy	Gate boundaries were set according to control samples (FMO-fluorescence minus one or isotype controls).

- [x] Tick this box to confirm that a figure exemplifying the gating strategy is provided in the Supplementary Information.
